# *FGFR1* tyrosine kinase domain duplication in pilocytic astrocytoma with anaplasia

**DOI:** 10.1101/mcs.a002378

**Published:** 2018-04

**Authors:** Leomar Y. Ballester, Marta Penas-Prado, Norman E. Leeds, Jason T. Huse, Gregory N. Fuller

**Affiliations:** 1 Department of Pathology and Laboratory Medicine and Department of Neurosurgery, University of Texas Health Science Center, Houston, Texas 77030, USA;; 2 Department of Neuro-Oncology, The University of Texas MD Anderson Cancer Center, Houston, Texas 77030, USA;; 3 Department of Diagnostic Radiology, Section of Neuroimaging, The University of Texas MD Anderson Cancer Center, Houston, Texas 77030, USA;; 4 Department of Pathology, Section of Neuropathology, The University of Texas MD Anderson Cancer Center, Houston, Texas 77030, USA

**Keywords:** astrocytoma

## Abstract

We report the case of a 27-yr-old male with visual field loss who had a 4.9-cm complex cystic mass in the right occipital lobe. Histologic examination showed pilocytic astrocytoma (PA) with anaplasia, and molecular characterization revealed *FGFR1* duplication with additional variants of unknown significance in several genes (*ARID1A, ARID1B, CHEK2, EPHA5,* and *MLL2*). This is one of only a very few reported cases of anaplastic PA with characterization of molecular alterations.

## CASE

**Figure 1.**
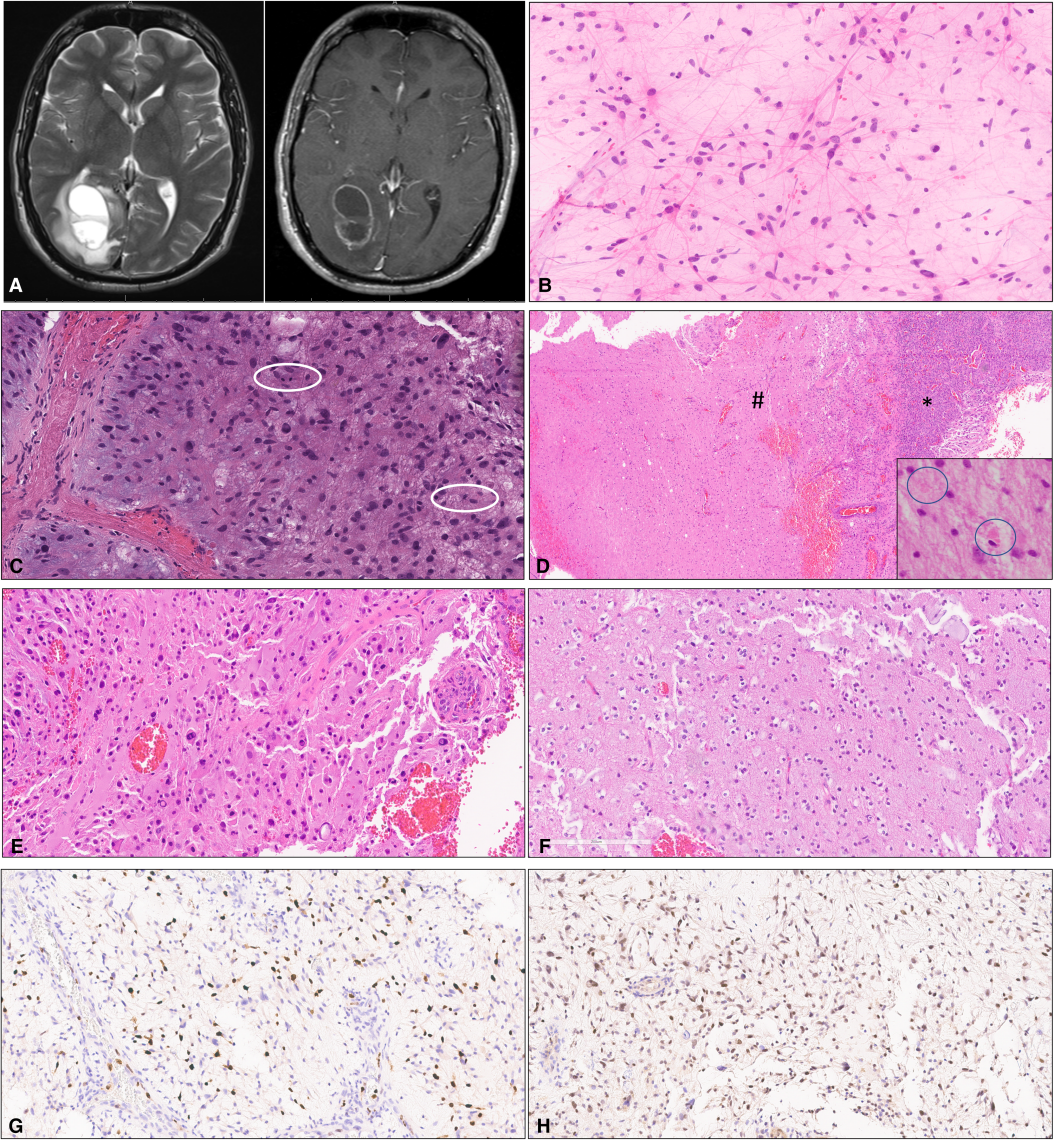
(*A*) Axial T2-weighted MRI sequence (*left*) and T1-weighted MRI sequence with contrast (*right*) showing the presence of a complex lesion in the right occipital lobe with ring enhancement. (*B*) Cytologic smear preparation showing neoplastic astrocytes with long, delicate bipolar cytoplasmic processes (pilocytic morphology). (*C*) Mitotic activity and myxoid background. (*D*) Arcade of vascular proliferation (*) and relatively sharp demarcation with adjacent brain parenchyma (#). Rare eosinophilic granular bodies (EGBs) were present (*inset*). (*E*) Hypercellular brain parenchyma with pleomorphic tumor cells. (*F*) Tumor areas with myxoid appearance. (*G*) Ki67 antigen (MIB1) immunostain showing elevated labeling in the region with anaplasia. (*H*) Expression of p53 protein by tumor cells in the region with anaplasia.

A 27-yr-old male presented to the emergency department with a complaint of left visual field loss after 2 mo of intermittent blurry vision. On examination, he had left homonymous hemianopsia. Magnetic resonance (MR) imaging demonstrated a 4.9 × 2.7 × 2.5-cm complex multiloculated ring-enhancing lesion in the right occipital lobe with hemorrhagic foci (Fig. 1A). Right parieto-occipital lobe craniotomy with gross total resection of the tumor was performed.

Microscopic examination of the tissue showed a glial neoplasm with atypical cells displaying thin bipolar cytoplasmic processes (Fig. 1B), focally elevated mitotic activity (Fig. 1C), and prominent arcades of vascular proliferation that correlated with prominent ring enhancement seen on preoperative MR imaging (Fig. 1D). Tumor cell morphology varied from spindled to pleomorphic (Fig. 1E), with features of pilocytic astrocytoma (PA) and pleomorphic xanthoastrocytoma (PXA). Much of the tumor exhibited a prominent myxoid background (Fig. 1C,F); focal hyalinization of blood vessels was also observed. A relatively circumscribed interface with brain parenchyma was identified in several tissue fragments (Fig. 1D). Rosenthal fibers were not identified but EGBs were present (inset). Elevated mitotic activity was present (up to 7 mitoses per 10 high-power fields), and quantitative analysis yielded a correspondingly elevated Ki67 antigen (MIB1) labeling index (Fig. 1G).

Immunophenotyping showed focal areas of strong nuclear staining for p53 protein (Fig. 1H). Molecular profiling of the tumor was performed using a next-generation sequencing assay that interrogates 315 genes for mutation, as well as the introns of 28 genes involved in gene rearrangements (FoundationOne assay, Foundation Medicine, INC). A genomic alteration involving duplication of exons 10–18 of the *FGFR1* gene was identified. In addition, five variants of unknown clinical significance were also identified (Table 1). Integration of the clinical, histologic, and molecular information available led to a final diagnosis of PA with anaplasia. Treatment with concurrent radiation and temozolomide was initiated.

**Table 1.** Variants detected by next-generation sequencing analysis of tumor tissue

Gene	Chromosome	Position	Variant type	Predicted effect
*FGFR1*	8p11.23	Exons 10–18	Duplication	Pathogenic
*ARID1A*	1p36.11	p.P21_S22INSPP	Insertion/deletion	VUS
*ARID1B*	6q25.3	p.C251G	Missense mutation	VUS
*CHEK2*	22q12.1	p.T367fs*15	Frameshift mutation	VUS
*EPHA5*	4q13.2	p.D348G	Missense mutation	VUS
*MLL2*	12q13.12	p.E5292D	Missense mutation	VUS

VUS, variant of unknown significance.

## TECHNICAL ANALYSIS

Molecular profiling of the tumor was performed using a next-generation sequencing assay that interrogates 315 genes for mutations, as well as the introns of 28 genes involved in gene rearrangements (FoundationOne assay, Foundation Medicine, INC). The test performs at a median depth of coverage of 500×.

## DISCUSSION

The diagnostic challenges associated with this case relate to the presence of histologic features that overlap between PXA and PA and the increased mitotic activity with vascular proliferation, focally elevated Ki67 index, and p53 staining in a young patient. PA occurs in adults, with lobar localization being a frequent finding (∼44% in some series) (Stüer et al. 2007). However, PA with anaplastic features at initial presentation is uncommon (Stüer et al. 2007; Azad et al. 2009). Anaplasia in PA has been associated with shortened survival (Rodriguez et al. 2010b). The current WHO 2016 Classification of Tumors of the Central Nervous System recognizes the existence of PA with anaplasia; however, specific grading criteria for this entity have yet to be defined (Louis et al. 2016).

Mitogen-activated protein kinase (MAPK) pathway alterations are common in PA; in fact, PA is considered to be predominantly a single pathway disease resulting from MAPK dysregulation (Jones et al. 2013; Pathak et al. 2017). PXA and PA were considered in the differential diagnosis of this case, although the classic genetic alterations present in PXA or PA (BRAF p.V600E mutation and *BRAF-KIAA1549* fusion, respectively) were not present. However, an *FGFR1* duplication, involving exons 10–18, which contains the protein tyrosine kinase domain (TKD), was identified. Duplication of the *FGFR1* TKD has been reported in low-grade astrocytomas (including PA), usually in an extracerebellar location, and in dysembryoplastic neuroepithelial tumor (DNET) (Jones et al. 2013; Zhang et al. 2013; Rivera et al. 2016; Fina et al. 2017). In one study, 24% of diffuse WHO 2007 grade II cerebral gliomas showed a duplication of the *FGFR1* TKD. *FGFR1* TKD duplication leads to autophosphorylation and activation of the MAPK/ERK and the PI3K pathways and has been shown to drive tumorigenesis (Zhang et al. 2013). The overall histologic features of this case are in favor of PA, and under the current classification system, this tumor is best classified as an anaplastic PA. Nonetheless, we recognize the possibility that the group of tumors with *FGFR1* TKD duplication could represent a molecularly distinct subtype of glioma. However, further studies are required to confirm this assertion.

In addition to the *FGFR1* TKD duplication, this case of PA with anaplasia showed additional genetic alterations of unknown biological and clinical significance (Table 1). The COSMIC, ExAC, and ClinVar databases were used to evaluate the potential significance of the additional variants detected in this case of PA with anaplasia. None of the variants is reported in the ExAC database. There is only a single entry of the *ARID1A* p21_S222INSPP variant in ClinVar (reported as likely benign), but we consider the evidence insufficient to make a final determination. There is one COSMIC entry for the *CHEK2* T367fs*15 variant, and ClinVar shows 14 submissions of frameshift mutations in *CHEK2*, starting at codon 367. The variant is considered likely pathogenic in ClinVar. However, the significance of this variant in the context of our case is uncertain.

Genetic defects in *ARID1A*, *ARID1B*, and *CHEK2* have been reported in brain tumors (Sallinen et al. 2005; Jones et al. 2012; Forbes et al. 2015; Park et al. 2015). It remains to be elucidated whether or not the *FGFR1* duplication, or the additional genetic defects identified in this case, plays a direct role in the development of anaplastic features in PA. The molecular defects associated with PA have been characterized and primarily involve the MAPK pathway (e.g., *BRAF-KIAA1549* fusion, BRAF p.V600E). However, the molecular defects associated with anaplasia in PA are not well known, although an association with neurofibromatosis type 1, the PI3K pathway, and p16 loss have been reported (Rodriguez et al. 2010a; Hsieh et al. 2012; Yeo et al. 2013). This case is one of only very few reports addressing the molecular defects involved in PA with anaplasia (Rodriguez et al. 2010a) and highlights the potential involvement of *FGFR1* TKD in anaplastic PA.

## ADDITIONAL INFORMATION

### Data Deposition and Access

The variants were submitted to ClinVar (https://www.ncbi.nlm.nih.gov/clinvar/) and can be found under accession numbers SCV000692539, SCV000692540, SCV000692560, and SCV000692561.

### Ethics Statement

The study was performed with approval of the Institutional Review Board (IRB) at the University of Texas MD Anderson Cancer Center (# PA17-0216) with waiver of informed consent.

### Competing Interest Statement

The authors have declared no competing interest.

### Referees

Mariarita Santi

Kar-Ming Fung

Anonymous
